# Unraveling chemotherapy-evoked hepatic dysfunction: a deep dive into cyclophosphamide-related liver injury

**DOI:** 10.1007/s00210-025-04583-0

**Published:** 2025-09-27

**Authors:** Ehab E. Sharata, Mina Ezzat Attya, Marwa M. Khalaf, Remon Roshdy Rofaeil, Ramadan A. M. Hemeida, Amira M. Abo-Youssef

**Affiliations:** 1 Department of Pharmacology & Toxicology, Faculty of Pharmacy, Deraya University, Minia, 61111 Egypt; 2 Department of Pathology, Faculty of Medicine, Minia University, Minia, 61519 Egypt; 3 Department of Pharmacology & Toxicology, Faculty of Pharmacy, Beni-Suef University, Beni-Suef, 62514 Egypt; 4 Department of Medical Pharmacology, Faculty of Medicine, Minia University, Minia, 61519 Egypt

**Keywords:** Liver injury, Cyclophosphamide, Apoptosis, Inflammation, Oxidative stress

## Abstract

Cyclophosphamide (CPA) is an alkylating drug utilized in the treatment of several cancers and autoimmune illnesses. Liver injury is a serious adverse effect linked to the administration of CPA. Nonetheless, the mechanism behind this toxicity remains incompletely elucidated; mechanistic investigations have identified oxidative stress, inflammatory responses, and apoptosis as pivotal elements contributing to CPA-induced liver dysfunction. In addition, CPA triggers the production of reactive oxygen species that act as damage-associated molecular patterns that rapidly activate TLR4/MYD88/NF-κB and NLRP3 inflammasome signaling cascades. Additionally, Nrf2/HO-1, α-klotho, and P-AMPK, which have anti-inflammatory and antioxidative characteristics, are thought to be important signaling pathways that mitigate oxidative stress in CPA-induced liver dysfunction. This review comprehensively covers all aspects of liver injury, including its epidemiology of drug-induced liver injury, risk factors, clinical presentation, chemotherapy-induced liver injury severity index, pathogenesis of CPA-induced liver injury and molecular mechanisms, and therapeutic choices. This study seeks to consolidate all known data about CPA-evoked liver injury, focusing on the probable redox molecular pathways underlying CPA-induced liver injury and recent drugs that showed a protective impact. In conclusion, studying these molecular pathways might open the way for early alleviation of hepatic dysfunction.

## Chemotherapy-induced liver injury

Chemotherapy-induced liver injury (CILI) is a significant clinical concern that complicates cancer treatment and may lead to interruptions or dose reductions, reducing long-term therapeutic efficacy (Li et al. 2025). Hepatotoxicity rates among patients receiving chemotherapy vary widely by drug and clinical setting. For example, A previous study reported that up to 85% of patients undergoing systemic chemotherapy can develop liver steatosis, with more severe outcomes like steatohepatitis, particularly dangerous when accompanied by elevated bilirubin (Ramadori and Cameron 2010). In clinical practice, chemotherapy drugs such as methotrexate, cisplatin, and cyclophosphamide are among the most frequent causes (Dass 2020). Many of these medications need metabolic bioactivation by the liver, rendering it particularly susceptible to the harmful effects of their metabolites (Corsini and Bortolini 2013).

## Risk factors of chemotherapy-induced liver injury

Some of the risk factors associated with CILI include:

- Old age: The accepted opinion was that those above the age of 55 were more likely to suffer from liver dysfunction due to impaired liver function (Devarbhavi 2012).
- Gender: CILI is typically associated with women. The majority of patients were female, according to studies conducted in Sweden (56% of all cases) and Japan (58% of all cases). There is no established cause; however, it might be related to the greater rates of breast cancer and chemotherapy exposure in women (Björnsson and Olsson 2005; Takikawa et al. 2009).
- Alcohol: Glutathione reserves are depleted by chronic alcohol consumption, especially when combined with undernutrition (Chalasani et al. 2008).
- Multiple drugs: The complicated and hard interaction between medications given simultaneously is a real concern. When two medications interact in a certain way, the risk of hepatotoxicity from one drug rises when the second drug is taken. For example, the use of azathioprine increases the risk of cyclophosphamide-associated hepatotoxicity (Floyd et al. 2006).
- Nutrition: According to reports among individuals with human immunodeficiency virus or alcoholism, nutritional deficiencies might increase the risk of liver dysfunction. Low glutathione levels in these individuals are thought to be responsible for this tendency. Hypoalbuminemia is an alternative indicator for malnutrition, providing indirect evidence to support this theory (Singla et al. 2010). Excessive intake of herbal and multi-ingredient nutritional supplements, including anabolic steroids and green tea extract, has been linked to liver damage because of their complex and occasionally unknown ingredients (Navarro et al. 2017). Additionally, nutritional status or diet composition, like high-fat diets, can alter liver enzyme activity (such as CYP enzymes) and affect drug metabolism, which may increase susceptibility to liver injury (Chen et al. 2015).
- Patient health status and the presence of pre-existing diseases: Patient health status and the presence of pre-existing diseases are crucial risk factors for liver injury during chemotherapy administration. Individuals with underlying chronic liver diseases such as hepatitis B or C, cirrhosis, or nonalcoholic fatty liver disease have a heightened susceptibility to hepatotoxicity due to reduced hepatic reserve and altered drug metabolism. Chemotherapy can exacerbate these conditions, sometimes leading to progressive liver dysfunction or even acute liver failure. For example, methotrexate can accelerate fibrosis in patients with fatty liver disease (Li et al. 2025; Atallah et al. 2023). Additionally, other comorbidities such as diabetes, obesity, and cardiovascular disease further increase hepatic vulnerability. Therefore, assessing baseline liver function and comorbid health conditions is essential before starting chemotherapy to inform the choice of agents, determine dose adjustments, and implement closer monitoring to minimize the risk of serious liver injury (García-Cortés and García-García 2022).
- The dose and route of chemotherapy administration: Both dose and route of administration are significant risk factors for liver damage. The risk of serious liver consequences, such as steatosis, steatohepatitis, and veno-occlusive disease, can be raised by high-dose chemotherapy, particularly when employed in aggressive cancer regimens or pre-transplant cytoreduction. Dose modifications are often necessary to prevent progression to irreversible liver damage. The risk also varies based on the type of chemotherapeutic drug and its individual hepatotoxicity profile (Ramadori and Cameron 2010). The pattern and risk of liver damage are greatly influenced by the route of administering chemotherapy. When chemotherapeutic drugs are administered intravenously, they enter the bloodstream immediately and frequently reach the liver in larger amounts more quickly. This can result in acute hepatic toxicities such as fulminant liver failure and veno-occlusive disease, especially when using drugs that are known to have hepatotoxic potential (Floyd et al. 2006; Allard et al. 2015).

## Effect of chemotherapy on liver function: hepatocellular and clearance function.

Serum aminotransferase elevation is a common occurrence during and after cytotoxic treatment. Liver steatosis, which develops in as Many as 85% of patients, is an indicator of improper lipid metabolism due to altered lipoprotein production in the liver cells. Vulnerability to irreparable hepatocellular damage increases when hepatic lipid content rises, leading to the increased inflammatory cells that may be recruited, especially during subsequent rounds of chemotherapy (Ramadori and Cameron 2010; Teoh and Farrell 2006). Disruption of the supply of Kupffer cell precursor cells to the liver occurs after chemotherapy that suppresses bone marrow cell replication. Moreover, in the intestines, chemotherapy also inhibits the replication of epithelial cells. Hyperpermeability of the gastrointestinal tract causes an influx of harmful and infectious substances into the liver, which in turn accelerates the turnover of macrophages in the liver. The likelihood of infection may rise as a consequence of this divergence (Ramadori and Cameron 2010).

Hepatotoxicity from chemotherapy can manifest in a wide range of ways, from mild symptoms like an increase in liver enzymes without any noticeable symptoms, jaundice, and cholestasis to more severe complications like advanced fibrosis, malignant transformation, sinusoidal obstruction (which manifests as weight gain, ascites, and tender hepatomegaly), and finally, fulminant hepatic failure. Elevated levels of serum aminotransferases, alkaline phosphatase, and bilirubin indicate the extent of liver damage (Bahirwani and Reddy 2014).

The degree of chemotherapy-induced liver dysfunction can be graded using the following criteria established by the National Cancer Institute based on the levels of alanine aminotransferase (ALT), aspartate aminotransferase (AST), alkaline phosphatase (ALP), and bilirubin (Grigorian and O'Brien 2014).

**Table:** 

Grade 1 (mild)	ALT/AST/ALP > ULN (upper limit of normal) to 2.5 × ULN; bilirubin > ULN to 1.5 × ULN
Grade 2 (moderate)	ALT/AST/ALP > 2.5 to 5 × ULN; bilirubin > 1.5 to 3 × ULN
Grade 3 (severe)	ALT/AST/ALP > 5 to 20 × ULN; bilirubin > 3 to 8 × ULN
Grade 4 (life-threatening)	ALT/AST/ALP > 20 × ULN; bilirubin > 8 × ULN

## Diagnostic criteria for chemotherapy-induced elevated liver enzymes

The tumor’s toxic consequences, such as hepatic metastases, preexisting liver disease, effects of co-medication, and infections, must be considered while making a differential diagnosis of increased liver enzymes after chemotherapy. To ascertain the likelihood that drug-induced liver damage has occurred, a patient evaluation is required, with the following steps (Ramadori and Cameron 2010; Floyd et al. 2006):

- How long does it take for liver enzymes to rise once a medicine is given?
- Resolution rate after discontinuation
- Rule out other explanations (such as alcohol, biliary tract illness, ischemia, or viral hepatitis)
- Medicines taken at the same time
- Tracking the records of comparable cases

## Risk of liver dysfunction with alkylating agents

Apart from cyclophosphamide and ifosfamide, alkylating drugs are often not considered hepatotoxic and can be safely administered even when there is underlying liver insufficiency, without the need to reduce dosage (Bahirwani and Reddy 2014). The theory behind how these drugs harm the Liver is that they deplete glutathione and cause oxidative damage. There has been a 3% documented incidence of hepatotoxicity connected with ifosfamide. There have been reports of people experiencing cyclophosphamide liver damage along with elevated aminotransferases (El-Gendy et al. 2022; Subramaniam et al. 2013).

### Cyclophosphamide

**Fig. 1:**
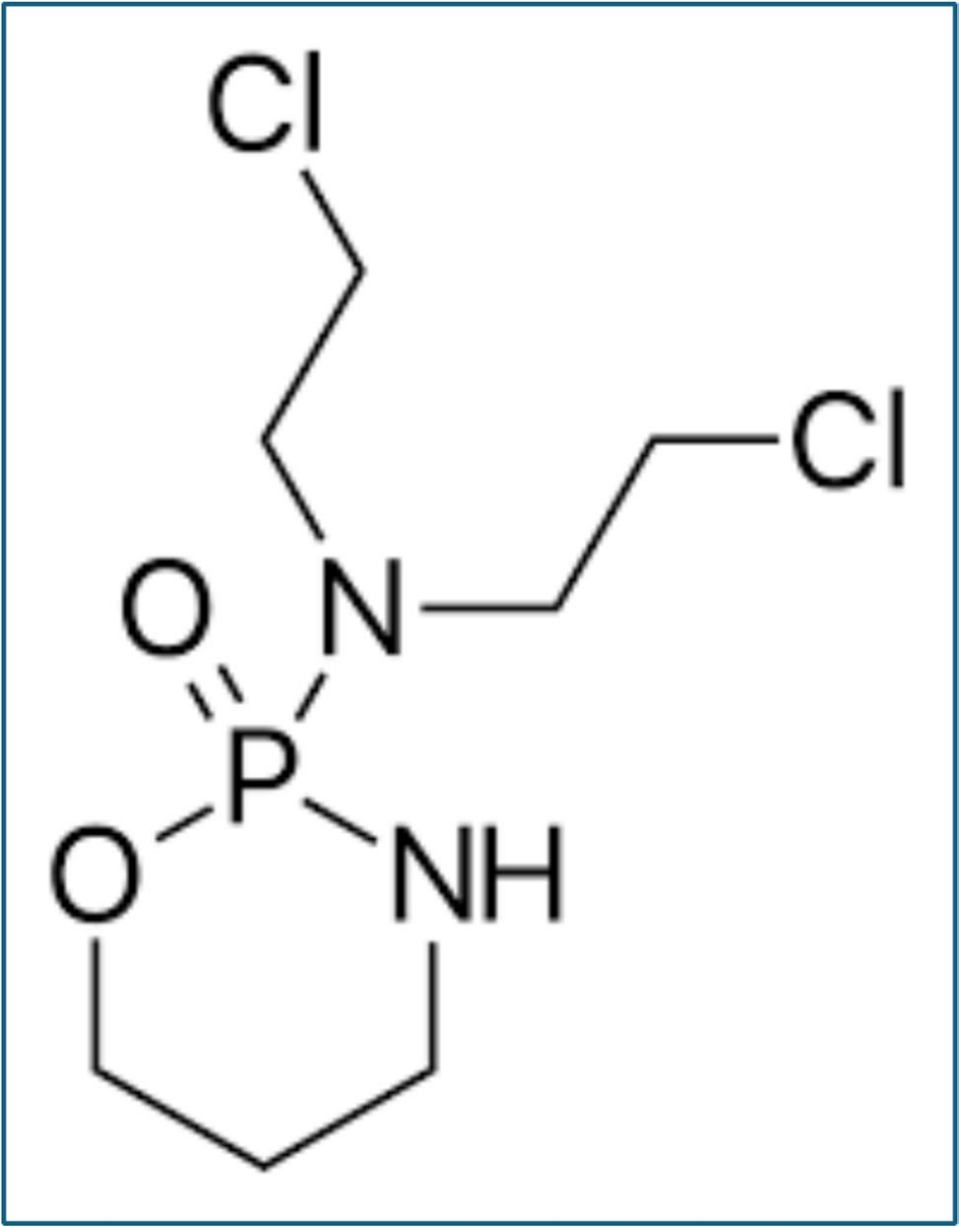
Cyclophosphamide structure (Nascimento et al. 2020)

A potent anti-cancer medication is cyclophosphamide (Fig. 1). Half a century after its discovery, CPA is still often utilized to treat a variety of cancers and autoimmune diseases. After preliminary clinical trials of CPA for cancer therapy began in 1958, the medicine was approved by the Food and Drug Administration (FDA) in 1959 as the 8th antineoplastic agent. Additionally, it is authorized for the treatment of pediatric nephrotic syndrome. Despite widespread use, it has never been officially approved for use in autoimmune diseases (Emadi et al. 2009).

### Pharmacokinetics

#### Dosage and administration

The recommended daily dosage of CPA for immunosuppressant and cancer therapy purposes is 100 to 200 mg taken orally. In the treatment of certain Malignancies, greater doses, ranging from 600 to 1000 mg per m^2^, are frequently given intravenously every 3 to 4 weeks (Moore et al. 1988). Because the liver must activate CPA before it can produce cytotoxicity, its intra-arterial injection is ineffective (Lokich and Bothe 1984; Jonge et al. 2005).

#### Absorption

Oral CPA reaches its Maximum absorption and concentration after 1 h. Area under the curve ratio following oral vs intravenous delivery of the medication ranges from 0.87 to 0.96 (Wagner and Fenneberg 1984). The initial pass through the liver involves the metabolism of some of the taken medicine. Because CPA is activated throughout this stage, the drug Maintains systemic accessibility and achieves a true bioavailability close to 100% (Moore 1991).

#### Distribution

About 20% of CPA is bound to proteins, and this binding is independent of dosage (Moore 1991). About 30 to 50 L is the predicted volume of distribution of CPA, which closely corresponds to the total quantity of body water (Jonge et al. 2005).

#### Metabolism

**Fig. 2:**
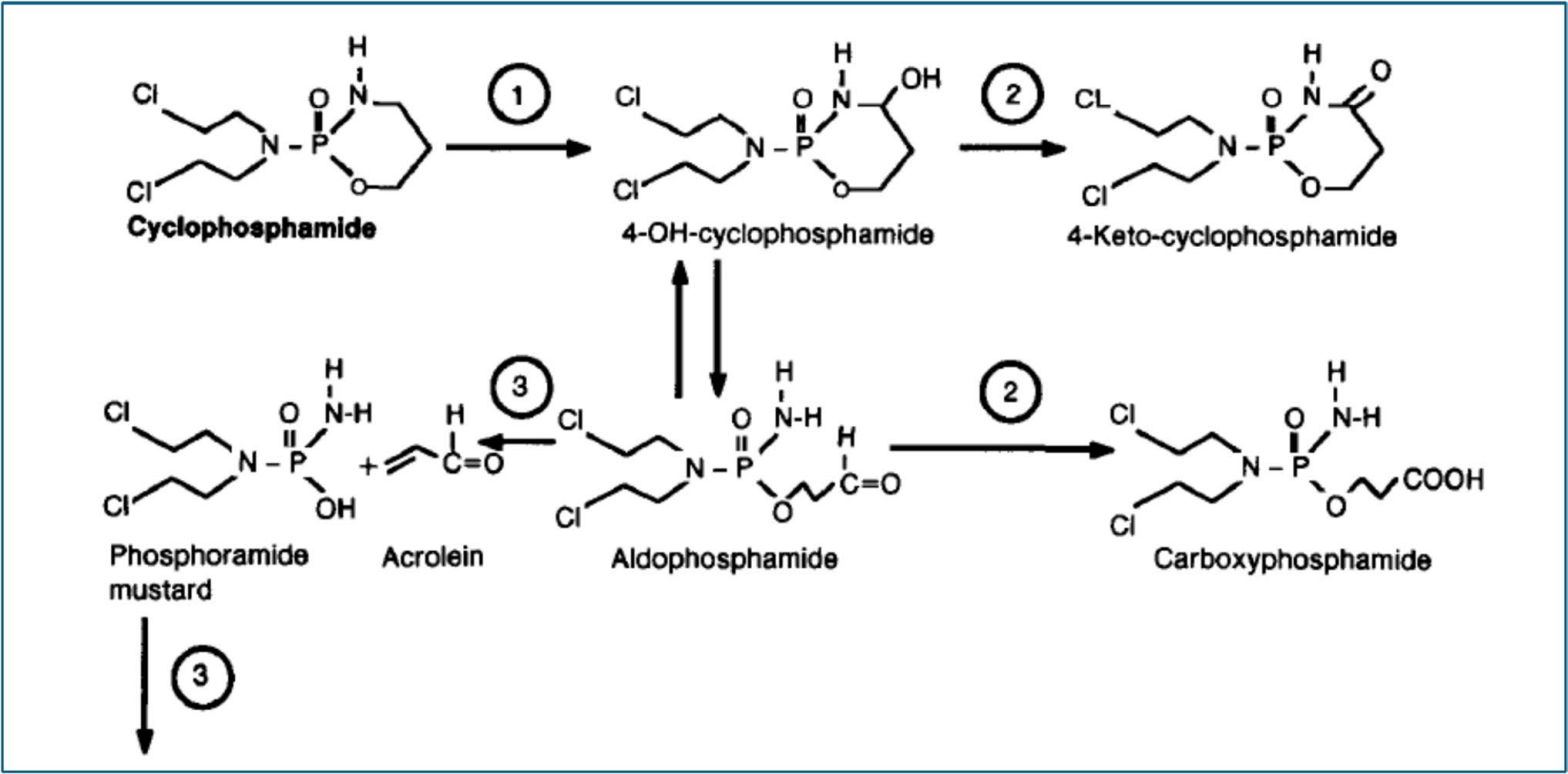
Metabolism of cyclophosphamide (Moore 1991)

Inactive CPA is converted to active 4-OHCPA by hepatic microsomal oxidases; 4-OHCPA and its aldophosphamide metabolite are then in a state of equilibrium. Inactive metabolites, ketocyclophosphamide and carboxyphosphamide, can be produced from 4-OHCPA and aldophosphamide by aldehyde dehydrogenases. These enzymes are present in many different types of tissues, including cancer cells (Ataya et al. 1990). The formation of acrolein and phosphoramide mustard from aldophosphamide can also occur spontaneously through a process that is facilitated by albumin, bases, and certain biological enzymes. Phosphoamide mustard has a 40–50 min half-life inside cells before it spontaneously hydrolyzes (Boyd et al. 1986). The breakdown of 4-OHCPA to phosphoramide mustard produces acrolein, a highly reactive aldehyde that may enhance CPA-induced cellular damage by lowering cellular glutathione levels (Jonge et al. 2005; Moore 1991). As demonstrated in Fig. 2.

#### Excretion

The kidneys filter out almost all of the CPA and its metabolites in the first 24 h of treatment (Sladek et al. 1980). The percentage of unaltered dose excreted in urine is just 20%. CPA or its metabolites, of which carboxyphosphamide is the primary metabolite, are excreted in the urine at levels ranging from 30 to 60% of the total dose (Hadidi et al. 1988). However, according to Chan et al. the main metabolite in urine is phosphoramide mustard (Chan et al. 1994). The elimination of a small fraction of the CPA dose occurs through feces and inhaled air (Jonge et al. 2005).

#### Pharmacodynamics

Cyclophosphamide exerts its effects by alkylating DNA. The medicine is metabolized into an active form that inhibits protein synthesis via DNA and RNA crosslinking, irrespective of the cell cycle phase (Mills et al. 2019; Korkmaz et al. 2007). The phosphoramide metabolite forms intra- and inter-strand cross-links at the guanine N-7 position. Programmed cell death is the end outcome of these permanent changes (Colvin 1999). Acrolein is the primary cause of hemorrhagic cystitis, even though it does not have any anticancer effects. CPA has anti-cancer effects, but it also has immunosuppressive characteristics and a T cell-specificity. The medication decreases the release of interferon-gamma and IL-12 (Chatelanat et al. 2018; Ahlmann and Hempel 2016).

#### Clinical indications

The main indication for cyclophosphamide’s approval is for the treatment of advanced malignant lymphomas, such as multiple myeloma, non-Hodgkin lymphoma, and Hodgkin lymphoma (Mills et al. 2019; Korkmaz et al. 2007). The FDA has also approved CPA for the treatment of ovarian cancer, nephrotic syndrome in children, disseminated neuroblastoma, and breast cancer (Colvin 1999). As an effective immunosuppressive medication, CPA has been demonstrated in several trials to be advantageous in the treatment of multiple sclerosis. To lessen the likelihood of transplant failure and graft-host complications, CPA has also been administered prior to transplantation (Emadi et al. 2009).

#### Adverse effects

Toxic effects on the bladder and gonadal organs are the most common adverse effects of cyclophosphamide (Martin et al. 1997). Several clinical investigations have shown common side effects of CPA use, including hemorrhagic cystitis, POI, hepatic dysfunction, myelosuppression, hair loss, nausea, and vomiting episodes (Dan et al. 2014). Chronic hemorrhagic cystitis can develop by using CPA without also hydrating properly or taking mesna at the same time. Because CPA causes myelosuppression, septic shock can develop (Dan et al. 2014). There have also been reports of pulmonary toxicity, secondary malignancies, myocarditis, pericardial effusion, and severe congestive heart failure (Atilla et al. 2020). Ovarian failure is a Major adverse effect of CPA, especially in women. In lupus patients using an intermediate dosage of CPA monthly, chronic amenorrhea is a Danger for 12% of women under the age of 25 and over 50% of women over the age of 30 (Emadi et al. 2009; Watson et al. 1985).

#### Contraindications

It is not recommended to use CPA in those who have severe reactions to the drug. The increased risk of hemorrhagic clot retention makes CPA an inappropriate choice for people with diseases that affect urine flow (Tabchi et al. 2019). Since CPA has been associated with negative effects on the growing fetus, pregnant women should not use the drug. Babies exposed to the CPA in breast milk are at increased risk for developmental delays, congenital abnormalities, and even death during fetal development.

## Pathogenesis of cyclophosphamide-evoked liver dysfunction

Hepatotoxicity associated with CPA has been well documented in clinical scenarios (Subramaniam et al. 2013; Zhu et al. 2015; Ming et al. 2019). Several pathways are implicated in CPA-induced liver injury.

### Role of oxidative stress in hepatic dysfunction induced by cyclophosphamide

During metabolism, the cytochrome P450 enzymes in hepatocytes convert the CPA into phosphamide mustard and acrolein (Qian et al. 2022; Aladaileh et al. 2019a). Prior research has indicated that liver damage caused by CPA is linked to acrolein (Fouad et al. 2016). Acrolein can cause the production and buildup of reactive oxygen species (ROS) in the liver, leading to the development of oxidative stress (OS) (Zhang et al. 2021a; Saleh et al. 2024). This will result in structural harm to the membranes of different organelles, such as the mitochondrial membrane in hepatocytes, and eventually lead to massive hepatocyte inflammation and programmed cell death (Mahmoud et al. 2017; Singh et al. 2018). The metabolic byproducts of CPA, such as acrolein free radicals, initiate a cascade of reactions that result in the oxidation of lipids, leading to excessive synthesis of MDA and a reduction in hepatic GSH content and SOD activity (Oyagbemi et al. 2016; Jiang et al. 2019). Several prior studies demonstrated that the pathophysiology of CPA-evoked liver dysfunction is associated with increased hepatic MDA content and decreased SOD and GSH levels (Temel et al. 2020; Sheweita et al. 2016).

### Role of inflammatory TLR4/MYD88/NF-κB/P38 MAPK pathway in hepatic dysfunction induced by cyclophosphamide

**Fig. 3:**
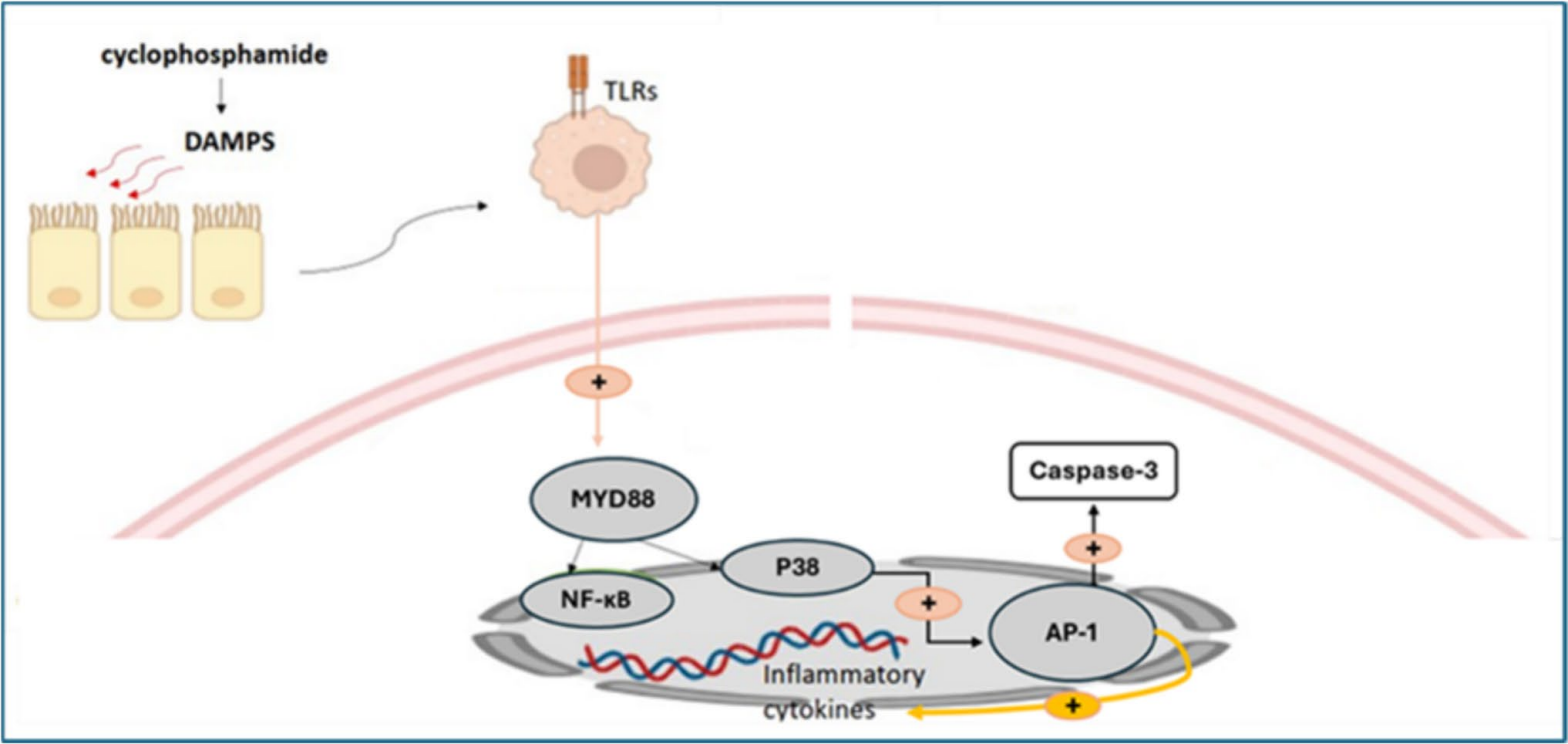
TLR4/MYD88/NF-κB/P38 MAPK signaling pathway

The TLR family includes Toll-like receptor 4 (TLR4), which is activated by ROS or damage-associated molecular patterns (DAMPs) (Zhai et al. 2004). When TLR4 on the cell membrane is triggered, TLR4 descending adaptor proteins, including myeloid differentiation primary response 88 (MYD88), are activated, and once the I*κ*B (inhibitor of nuclear factor-kappa B) complex is phosphorylated by activated MYD88, nuclear factor-kappa B (NF-κB) becomes functional as well (Khallaf et al. 2023; Abdelnaser et al. 2025a). The upregulation of NF-κB leads to enhanced production of pro-inflammatory cytokines, namely IL-1β, IL-18, and TNF-α (Fathy and Nikaido 2018; Aladaileh et al. 2019b; Zhang et al. 2021b; Mohyeldin et al. 2024). TLR4 also facilitates an alternate cascade that induces the activation of p38-mitogen-activated protein kinase (p38-MAPK), which subsequently initiates the inflammatory response and apoptotic death (Hassanein et al. 2023; Yan-Zi et al. 2018). MAPKs are serine-threonine kinases that specifically target proline residues (Haddad 2004). They function as transducers, relaying environmental stimuli to the nucleus. The p38-MAPK, a member of the MAPK family, is activated by OS, resulting in the buildup of ROS within cells and further modulating NF-κB and caspase-3 expression (Herlaar and Brown 1999; Chen et al. 2013). ROS produced under pathological conditions, such as exposure to CPA, act as signaling molecules that activate upstream kinases. Specifically, ROS triggers the activation of MAP kinase kinase kinases (MAP3Ks), such as ASK1 (apoptosis signal-regulating kinase 1). ASK1 phosphorylates and activates downstream MAP kinase kinases (MKK3/6), which in turn phosphorylate and activate p38-MAPK (Wu et al. 2023; Canovas and Nebreda 2021). Activated p38-MAPK can enhance ROS production by increasing the expression of pro-oxidant enzymes such as NADPH oxidase subunits and by impairing the activity of antioxidant defense enzymes, thus amplifying cellular oxidative stress (Rashed et al. 2011; Park et al. 2010; Hernandez et al. 2019). Furthermore, p38-MAPK can phosphorylate various intermediaries that facilitate the activation and nuclear translocation of NF-κB, enhancing the transcription of inflammatory markers such as TNF-α, IL-1β, and IL-6 (Saha et al. 2007; Ozbek et al. 2009). Activated p38-MAPK also plays a pro-apoptotic role by upregulating pro-apoptotic proteins and promoting the transcription and activation of caspase-3, a crucial executor of apoptosis (Chaparro-Huerta et al. 2005; Rofaeil et al. 2025; Mohyeldin et al. 2023). As graphically presented in Fig. 3. Multiple research findings concluded that the upregulation of the TLR4, p38 MAPK, and NF-κB signaling cascade is closely related to hepatic injury caused by CPA (Chen et al. 2019; Nafees et al. 2015; Mohammed et al. 2020; Tian et al. 2022).

### Role of apoptosis in hepatic dysfunction induced by cyclophosphamide

**Fig. 4:**
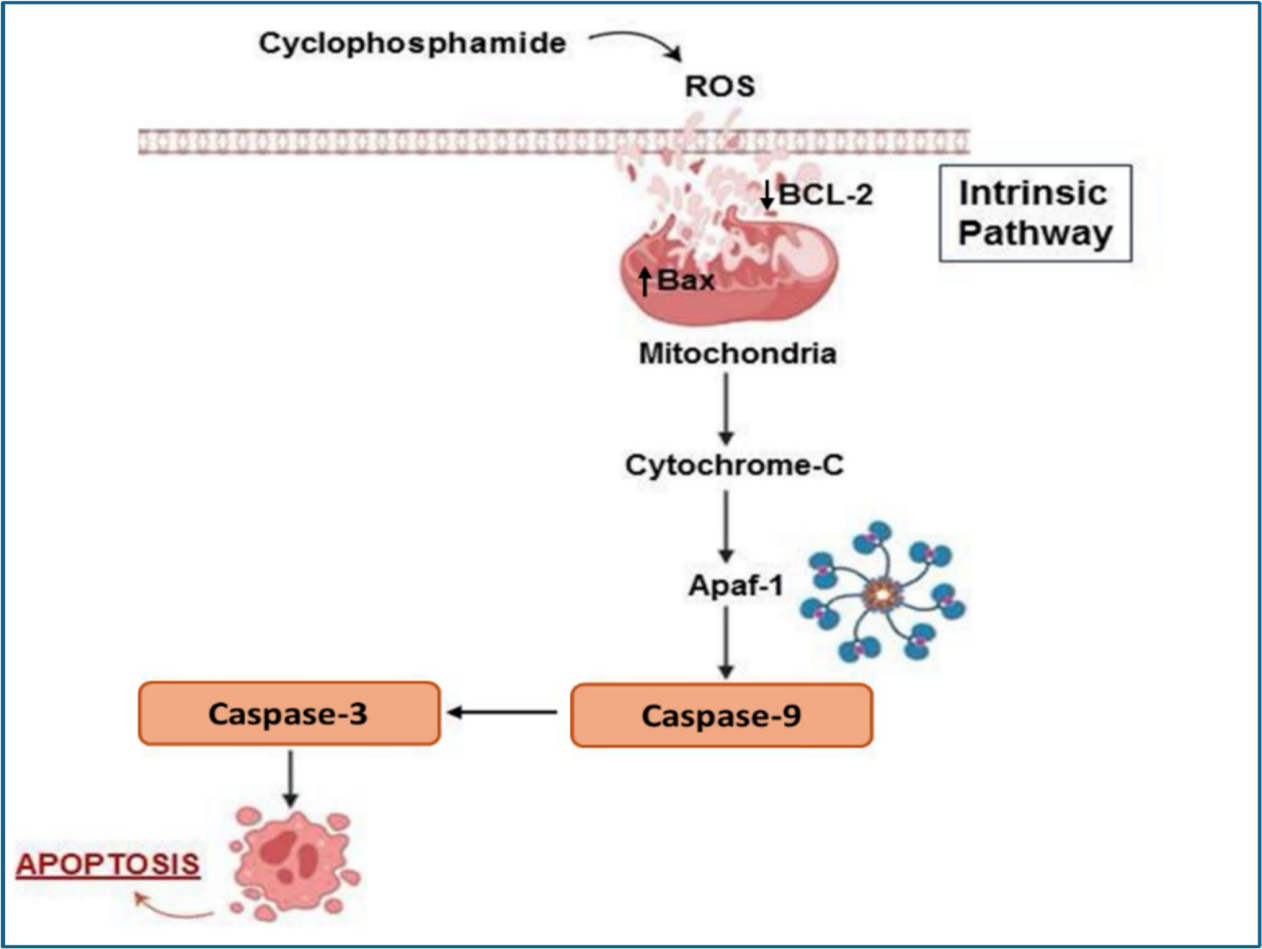
The intrinsic pathway of apoptosis

Cyclophosphamide induces apoptosis primarily through the intrinsic pathway by generating ROS, initiating a cascade of events that ultimately leads to mitochondrial outer membrane permeabilization (MOMP) (D’arcy 2019). Bcl-2-associated X (Bax) and B-cell lymphoma-2 (Bcl-2) are pro- and anti-apoptotic proteins, respectively, that tightly control the intrinsic pathway (Mohyeldin et al. 2025a). The key regulator of Bcl-2 gene expression is the transcription factor p53, which, when activated, enhances the production of Bax. Bax is triggered by ROS generation, leading to MOMP through the formation of pores. Cytochrome c escapes into the cytosol, where it interacts with pro-caspase 9 and apoptotic protease-activating factor-1 (APAF-1) to create the apoptosome. This ultimately leads to the activation of caspase 9, which then activates the effector caspase 3, resulting in apoptotic cell death (Kale et al. 2018; Aubrey et al. 2018; Mohyeldin et al. 2025b). As shown in Fig. 4, there is mounting evidence that apoptosis via the intrinsic pathway plays a critical role in the pathogenesis of CPA-induced hepatic dysfunction (Saleh et al. 2024). Multiple studies have shown that, alongside a marked reduction in hepatic Bcl-2 expression, CPA dramatically elevated the expression of hepatic Bax and caspase-3 (Zhao et al. 2022; Ibrahim et al. 2021; Famurewa et al. 2023).

### Role of NLRP3 inflammasome in hepatic dysfunction induced by cyclophosphamide

**Fig. 5:**
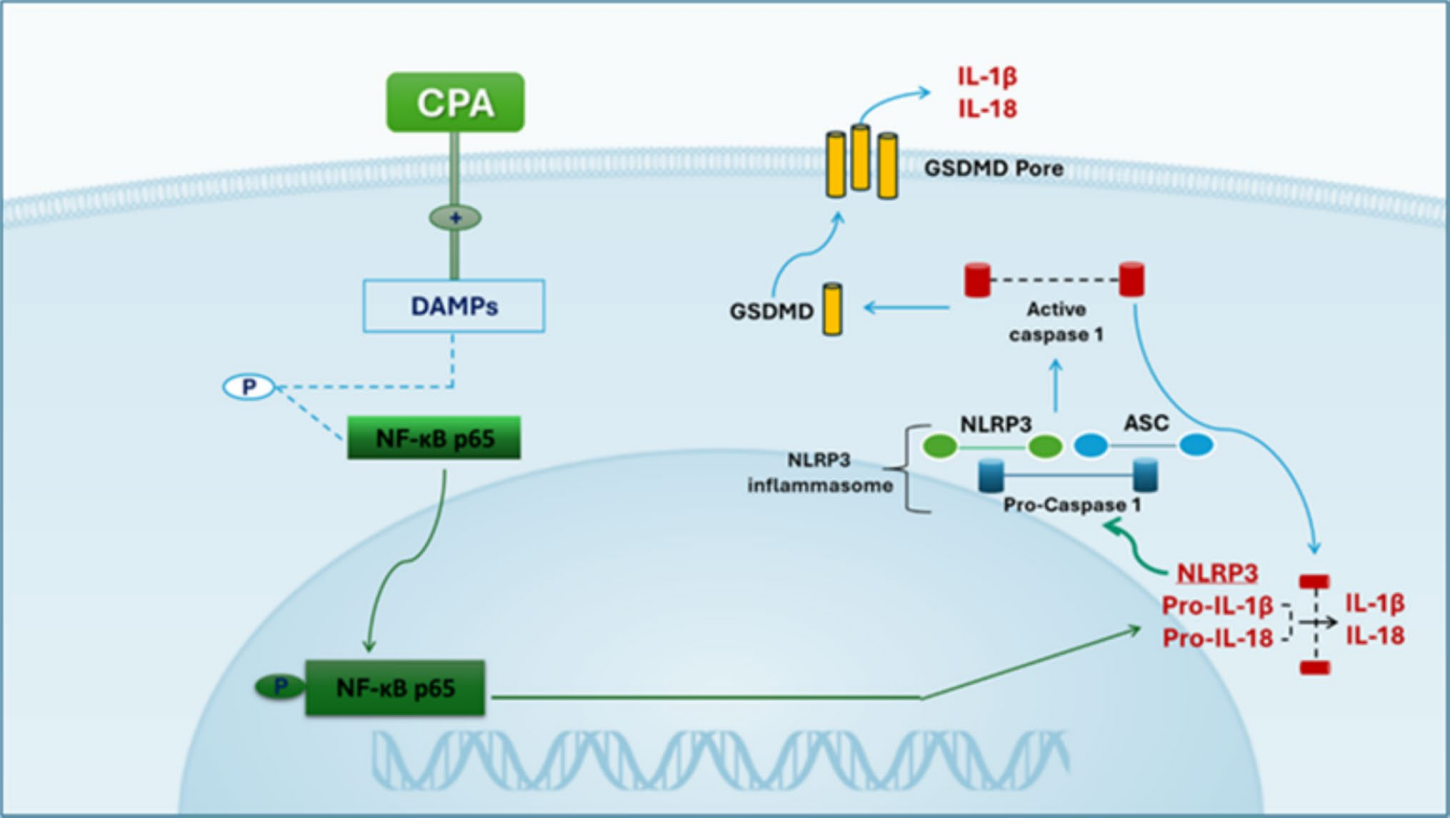
The NLRP3/Caspae-1/GSDMD signaling cascade

Inflammasomes are cytoplasmic multiprotein clusters that may be triggered by a wide range of endogenous and exogenous signals, such as ROS and DAMPs (Rofaeil et al. 2024). Multiple subtypes of inflammasomes have been identified. The nucleotide-binding oligomerization domain-like receptor family pyrin domain-containing 3 (NLRP3) inflammasome stands out among the rest (Zheng et al. 2020). In pyroptosis, cells produce proinflammatory mediators that lead to plasma membrane permeabilization, cellular enlargement, and eventual membrane rupture (Blevins et al. 2022). Defective mitochondria release an excessive amount of ROS, which are the most common DAMPs that quickly activate the NLRP3 (West and Shadel 2017). The inflammasome formation and pyroptosis molecular steps are as follows. To begin with, proinflammatory proteins such as pro-IL-1β, pro-IL-18, and NLRP3 are stimulated to be produced by NF-κB in response to ROS (Abdelnaser et al. 2025b). Furthermore, the NLRP3 inflammasome is formed by the assembly of procaspase 1, NLRP3, and apoptosis-associated speck-like protein (ASC). This leads to the activation of pro-caspase 1, which ultimately cleaves pro-IL-1β and pro-IL-18 into their final active forms. An additional mechanism by which activated caspase-1 enhances pyroptosis signaling is by hydrolyzing gasdermin D (GSDMD) and producing membrane pores (Kelley et al. 2019; Shi et al. 2015). As demonstrated in Fig. 5, several recent studies concluded that NLRP3 inflammasome activation contributes to hepatic dysfunction caused by CPA and accelerates pyroptotic death of hepatocytes in CPA-intoxicated rats (Ma et al. 2021; Mostafa et al. 2022; Mansour et al. 2017).

### Role of Nrf2/HO-1 pathway in hepatic dysfunction induced by cyclophosphamide

The critical sensor known as nuclear factor erythroid 2-related factor 2 (Nrf2) protects cells from xenobiotic-induced oxidative damage (Kensler et al. 2007). One redox-sensing system that Nrf2 provides is the encoding of antioxidants and detoxifying enzymes. A naturally occurring protein called Kelch-like ECH-associated protein 1 (KEAP1) inhibits Nrf2 function by preventing its proteasomal degradation (Taguchi et al. 2011; Alaaeldin et al. 2024). During xenobiotic exposure, the Nrf2/Keap1 pathway releases Nrf2, which then translocates into the nucleus and binds to the antioxidant response element sequences of genes, including heme oxygenase-1 (HO-1) and glutathione S-transferase (Ali et al. 2024). Nrf2 subsequently activates the protective antioxidant enzyme system, leading to a reduction in inflammation, OS, and hepatocellular injury (Al-Amarat et al. 2022). Numerous investigations have revealed that the downregulation of hepatic Nrf2/HO-1 is a key player in CPA-induced liver injury (Mahmoud et al. 2017; Sherif 2018). Furthermore, a lack of Nrf2 enhances the activation of NF-κB, resulting in aggravated stimulation of the NLRP3 inflammasome and the release of inflammatory cytokines (El-Agamy et al. 2018; Hou et al. 2018).

### Role of α-klotho in hepatic dysfunction induced by cyclophosphamide

A disease resembling accelerated aging can be caused by mutations in the aging suppressor gene “klotho,” which was discovered in 1997 (Kuro-o et al. 1997). The renal distal and proximal convoluted tubules are the most common sites of klotho production. New evidence also indicates that it is present in the liver, ovary, blood, urine, and cerebrospinal fluid, in addition to cardiovascular tissues (Kim et al. 2015; Khallaf et al. 2025). Within the klotho gene family, there are three subtypes: the most common, α-klotho, β-klotho, and γ-klotho (Olejnik et al. 2018). As a factor that prevents cellular death, fibrosis, and senescence, klotho promotes antioxidants and anti-inflammatory actions (Buchanan et al. 2020). A combination of factors, including an increase in OS and an elevation of inflammatory cytokines, can hasten the aging process and raise the likelihood of multisystem dysfunction when expression levels are low (Xu and Sun 2015). The circulating α-klotho functions as a hormone, exerting antioxidative stress, antisenescence, and antiapoptotic actions (Kim et al. 2013). Recently, studies have demonstrated a connection between hepatic impairment in non-alcoholic fatty liver disease, liver fibrosis, and circulating α-klotho deficiency (Chi et al. 2023; Liu et al. 2022). However, there is a shortage of studies exploring its specific antioxidant effects on CPA-induced hepatic oxidative damage. Furthermore, recent data corroborated the idea that upregulating α-klotho inhibited NLRP3-mediated pyroptosis (Li et al. 2019) and OS (Oh et al. 2015) while stimulating the Nrf2 signaling cascade (Xing et al. 2021). Recent evidence highlighted that levomilnacipran, a serotonin-norepinephrine reuptake inhibitor, offers significant hepatoprotective effects against CPA-induced liver injury. This protection is closely linked to the modulation of α-klotho expression and the suppression of the TLR4/p38-MAPK/NF-κB p65 pathway, as well as caspase-3-mediated apoptotic signaling. Notably, levomilnacipran was shown to upregulate α-klotho levels in hepatic tissue, which in turn attenuated the activation of inflammatory and apoptotic cascades triggered by CPA exposure. This suggests that agents capable of enhancing α-klotho expression might play a promising role in mitigating oxidative stress, inflammatory injury, and apoptosis in the liver during cyclophosphamide therapy (Sharata et al. 2025).

### Role of p-AMPK in hepatic dysfunction induced by cyclophosphamide

Adenosine monophosphate-activated protein kinase (AMPK) plays a vital role in a range of important cellular activities, including energy metabolism, anti-inflammatory responses, and the modulation of OS (El-Dessouki et al. 2024). It regulates many metabolic processes and is triggered when cellular energy levels drop, making it an essential player in keeping energy balance (Steinberg and Kemp 2009). Due to its presence in all cells, this kinase is essential for the regulation of several physiological functions. As a cellular energy sensor, AMPK measures the ratio of adenosine monophosphate (AMP) to adenosine triphosphate (ATP). In response to low ATP levels, which indicate energy depletion, AMPK is activated and endeavors to rectify the imbalance by enhancing pathways that generate energy (Steinberg and Hardie 2023). AMPK is Linked to several illnesses, especially those associated with metabolic dysfunction, including obesity, type 2 diabetes, and cardiovascular disease. Under these circumstances, insulin resistance, poor glucose uptake, and disturbed lipid metabolism may all be exacerbated by AMPK’s malfunction (Day et al. 2017). Lately, there has been an expanding array of research focusing on the role of AMPK in combating OS and liver impairment caused by chemotherapy (Bokhary et al. 2022; Xu et al. 2022). AMPK activation has been shown to upregulate Nrf2/HO-1 (Fischhuber et al. 2020) and downregulate the NLRP3 inflammasome via modulation of NF-κB (Abd El-Fattah et al. 2022).

## Therapeutic protection against CPA-evoked liver injury

As indicated in Table 1, several medications, both synthetic and natural, have shown promise in reducing CPA-induced liver dysfunction via a number of interrelated pathways, such as the suppression of oxidative stress, inflammation, and programmed cell death.

**Table 1:** Therapeutic protection against CPA-evoked liver injury in experimental models

Drug/agent	Experimental model	Method of induction	Protective mechanism	Reference(s)
**Arbutin**	• In vivo • Male rats	A single intraperitoneal injection of CPA (150 mg/kg)	Reduced inflammation, oxidative stress, and cell death via modulating the Nrf2/HO-1 pathway	Alruhaimi 2023
**Ginseng**	• In vivo • Male Sprague–Dawley rats	A single intraperitoneal injection of CPA (100 mg/kg)	Attenuated lipid peroxidation, regulated bile acid equilibrium, and restored glutathione and antioxidant enzymes	Zhu et al. 2015
**Fucoidan**	• In vivo • Male mice	CPA (80 mg/kg) once daily for 5 days	Augmented Nrf2/HO-1 expression, attenuated the TLR4/NF-κB signaling pathway, and reduced oxidative stress and inflammation	Tian et al. 2022
**Pyrroloquinoline quinone**	• In vivo • Male mice	CPA (80 mg/kg) for 5 consecutive days	It suppressed inflammation mediated by NF-κB and activated the antioxidant response mediated by Nrf2	Qian et al. 2022
**N-acetylcysteine**	• In vivo • Miniature pigs	CPA (50 mg/kg) for 2 weeks	Decreased TNF-α, mitigated oxidative stress, and lowered indicators of liver damage	Kang et al. 2020
**Quercetin**	• In vivo • Male Wistar albino rats	CPA (200 mg/kg) on day 10 of the experiment	Reduced MDA, ALT, ALP, AST; increased GSH; diminished oxidative stress, inflammation, and apoptosis	Doustimotlagh et al. 2020
	• In vivo • Female Wistar albino rats	CPA was given intraperitoneally at a dosage of 27 mg/kg once every three weeks for a total duration of ten weeks	Upregulated expression of the antioxidant parameters, such as SOD, reducing oxidative damage to the liver	Kocahan et al. 2017
**Capsaicin**	• In vivo • Male Wistar albino rats	CPA was administered intraperitoneally at a dosage of 200 mg/kg on the fourth day of treatment	Modulated oxidative stress, apoptotic signals, and cytokine pathways	Alam et al. 2023
**Boric acid**	• In vivo • Male Wistar albino rats	Rats were injected intraperitoneally with a single dose of CPA (200 mg/kg)	Suppressed inflammatory and apoptotic pathways	Cengiz et al. 2019; Önder et al. 2023
**Escin**	• In vivo •Male rats	A single intraperitoneal injection of CPA (200 mg/kg)	Attenuated oxidative damage, apoptosis, and lipid peroxidation	Cengiz et al. 2022
**Centella triterpene saponins**	• In vivo • Male Wistar albino rats	Rats received CPA (10 mg/kg/day, orally) for 30 days	Modulated oxidative stress, apoptotic signals, and inflammatory pathways	Duggina et al. 2015
**Resveratrol**	• In vivo • Male Sprague Dawley rats	CPA was given intraperitoneally (150 mg/kg)	Suppression of the NF-κB/TNF-α pathway, along with ensuing oxidative damage and inflammation	
**Chrysin**	• In vivo • Male Wistar albino rats	Rats received CPA (200 mg/kg/day) on the 7th day of the experiment	Reversed levels of inflammatory, apoptotic, and autophagic markers in liver tissues caused by CPA	Temel et al. 2020
**Atorvastatin**	• In vivo • Female Wistar albino rats	A single intraperitoneal injection of CPA (150 mg/kg)	The key mechanisms by which atorvastatin protects against CPA-induced hepatic damage are its antioxidant and anti-apoptotic properties	Hamzeh et al. 2018
**Levomilnacipran**	• In vivo • Male Wistar albino rats	A single intraperitoneal injection of CPA (200 mg/kg)	Modulating α-klotho/TLR4/p38-MAPK/NF-κB p65 and caspase-3-driven apoptosis	Sharata et al. 2025
**Trifluoperazine**	• In vivo • Male Swiss albino mice	A single intraperitoneal injection of CPA (200 mg/kg)	Reducing inflammatory responses, cell death, and oxidative stress in mice via regulating the Nrf2/HO-1 and AKT/mTOR-driven autophagy signaling pathways	Saleh et al. 2024
**Gallic acid**	• In vivo • Male rats	A single intraperitoneal injection of CPA (200 mg/kg)	Downregulated oxidative stress via its antioxidant activity	Oyagbemi et al. 2016
	• In vivo • Swiss albino mice	A single intraperitoneal injection of CPA (50 mg/kg)	Exhibited antioxidant and anti-inflammatory properties through upregulation of SOD and GSH	Shruthi and Shenoy 2021
**Blue berry**	• In vivo • Male Sprague Dawley rats	CPA was given intraperitoneally as a single dose (100 mg/kg)	Modulated TLR4/NF-κB and apoptotic pathways	Shi et al. 2014
**Allicin**	• In vivo • Male Sprague Dawley rats	A single intraperitoneal injection of CPA (200 mg/kg)	Blocking inflammatory and apoptotic mechanisms and activating Nrf2/ARE pathways	Sun et al. 2021
**Silymarin**	• In vivo • Male Wistar albino rats	A single intraperitoneal injection of CPA (100 mg/kg)	Exhibited antioxidant and anti-inflammatory properties	Ramadan and Abbas 2023
	• In vivo • Female Wistar albino rats	CPA was administered intraperitoneally at a dosage of 30 mg/kg for 7 days	Exhibited antioxidant properties and mitigated hepatotoxicity parameters	Avci et al. 2016
**Vitamin E**	• In vivo • Male albino rats	CPA was given as an intraperitoneal injection of 20 mg/kg/day for 2 weeks	Exhibits antioxidant, anti-apoptotic, and anti-inflammatory properties	Abdelfattah-Hassan et al. 2019
	• In vivo • Male Wistar albino rats	The rats received 20 mg/kg CPA once daily for 7 days	Improved the cell damage caused by increased oxidative stress due to CPA by enhancing the antioxidant capacity	Cuce et al. 2015
**Piracetam**	• In vivo • Wistar albino rats	A single intraperitoneal injection of CPA (200 mg/kg)	Amelioration of necroptosis, pyroptosis, and caspase-dependent apoptosis	Mostafa et al. 2022
**Taxifolin**	• In vivo • Swiss albino mice	CPA (30 mg/kg) was given for 10 consecutive days	Activation of the Nrf2/HO-1 pathway to mitigate inflammation and oxidative stress indicators	Althunibat et al. 2023
**Lutein**	• In vivo • Male Swiss albino mice	A single intraperitoneal injection of CPA (50 mg/kg)	Inhibition of ROS/NF-κB/P38 MAPK pathway	El-Kholy et al. 2017
**Berberine**	• In vivo • White male albino rats	A single intraperitoneal injection of CPA (200 mg/kg)	Modulating antioxidant status and inflammatory cytokines	Germoush and Mahmoud 2014
**Galangin**	• In vivo • Male Wistar albino rats	A single intraperitoneal injection of CPA (150 mg/kg)	Enhanced Nrf2 signaling and reduced inflammation, oxidative stress, and cell death	Aladaileh et al. 2019b
**Umbelliferone**	• In vivo • Male Wistar albino rats	A single intraperitoneal injection of CPA (150 mg/kg)	Upregulation of Nrf2 and PPARγ	Mahmoud et al. 2017

## Conclusion

The liver is an essential organ and is very vulnerable to chemotherapeutic agents, with cyclophosphamide-induced hepatic damage being a significant concern. Protective drugs have been facilitated by an enhanced comprehension of its etiology, encompassing oxidative stress, inflammation, and apoptosis. Consequently, more investigation is necessary to assess additional signaling molecular pathways involved in CPA-induced hepatic damage to develop novel therapies for alleviating liver injury produced by CPA. Additional clinical investigations are necessary to elucidate the potential applicability of the aforementioned protective agents as a supplemental therapy to prevent liver damage induced by CPA.

## Data Availability

All source data for this work (or generated in this study) are available upon reasonable request.
